# Development and Health System Deployment of an Electronic Health Record–Integrated Chatbot Intervention for Connecting Fall Risk Screening to Community Resources After Emergency Department Visits: Implementation Study

**DOI:** 10.2196/77237

**Published:** 2025-11-18

**Authors:** Audrey Keleman, Megan Bounds, Maxwell Lunt, Jennifer Portz, Bucky Ferozan, Jonathan Gomez Picazo, Kelly Bookman, Hillary D Lum, Elizabeth M Goldberg

**Affiliations:** 1 Eastern Colorado Geriatric Research, Education, and Clinical Center United States Department of Veterans Affairs Aurora, CO United States; 2 Physical Therapy University of Colorado Anschutz Medical Campus Aurora, CO United States; 3 Emergency Medicine University of Colorado Anschutz Medical Campus Aurora, CO United States; 4 Experience and Innovation UCHealth Aurora, CO United States; 5 Internal Medicine University of Colorado Anschutz Medical Campus Aurora, CO United States; 6 Emergency Medicine UCHealth Northern Colorado Fort Collins, CO United States; 7 Division of Geriatric Medicine Department of Medicine University of Colorado Anschutz Medical Campus Aurora, CO United States

**Keywords:** falls, older adults, digital health, referral pathway, high fall risk screening, fall prevention, emergency department

## Abstract

**Background:**

Emergency departments (EDs) routinely screen for fall risk, but patients are rarely notified of their results or referred to preventive resources. There is a critical need for an intervention that notifies patients when they are at risk for falls and automates referrals to fall prevention programs without increasing clinician workload. Chatbots can be used to provide patient education and community resources in a conversational, friendly manner. We developed and implemented an automated intervention using our health system's electronic health record (EHR) and an artificial intelligence chatbot, Livi, to address this gap in fall prevention across 17 EDs.

**Objective:**

This study aimed to share how we developed our fall risk notification and referral intervention and iteratively improved it based on end-user feedback*.*

**Methods:**

We collaborated with the EHR and ED operations teams to automate patient notification of fall risk and referral. First, we leveraged existing fall risk screening questions in nursing documentation to identify patients at risk for falls. We then developed an EHR workflow that delivers a QR code in the after-visit summary for all high-risk patients at ED discharge. Scanning the QR code launches a conversation with Livi, guiding users to physician-vetted, evidence-based, free or low-cost fall prevention resources in their area. In this workflow, only ED patients who are screened as high risk receive linkage to Livi, and clinicians do not need to manually place referrals or enter specific fall prevention resources at discharge. We conducted rapid, iterative usability testing of the Livi falls chatbot with 93 community members during the development process at 3 community fairs in distinct settings.

**Results:**

Rapid iterative testing led to enhancements in the intervention, such as increased font size, an option for Spanish language, additional geographic locations for fall prevention resources, home modification resources, the ability to self-assess for fall risk, fall prevention tips, and the ability for patients to leave feedback on the Livi chatbot. Because all EDs in the health system use the same instance of Epic, the EHR workflow was instantaneously deployed system-wide. The use of a QR code linked to the Livi chatbot also allows for the rapid updating of prevention resources.

**Conclusions:**

This study describes the formative development and system-wide implementation of the intervention. This scalable, EHR-integrated intervention demonstrates a novel and pragmatic approach to improving population health by capitalizing on existing clinical workflows and automating both risk notification and personalized resource referral for older adults without increasing clinician burden. The next steps include conducting a randomized controlled trial to assess the impact of the screening and referral tool on recurrent fall-related health care use compared with routine care in the ED. Formal evaluation of the implementation outcomes will be conducted in the planned trial.

## Introduction

Falls are the leading cause of injury-related emergency department (ED) visits among older adults [1]. Community-dwelling older adults who visit the ED after a fall have a 30% increased risk of falling again within 6 months compared to age-matched peers, and they often experience declines in functional abilities, such as balance, following discharge [2]. Evidence-based fall prevention programs can reduce fall risk by up to 30% [3,4], yet few older adults are successfully connected to these interventions after an ED visit. One persistent barrier is the absence of scalable digital health interventions to notify patients of their fall risk and refer them to appropriate, accessible programs.

Although ED nurses routinely screen older adults for fall risk, many patients are not informed of their screening results or provided with clear, actionable next steps [5,6]. Clinicians recognize the importance of fall prevention; however, acute medical needs typically take precedence during ED visits, limiting the time and attention available for preventive counseling [5,7]. Existing ED-based strategies have largely focused on automating risk identification using electronic health record (EHR) data [8] and manually referring high-risk ED patients to fall prevention clinics [9]. However, we are unaware of any intervention that can automate both patient notification of fall screening results and referral to fall prevention clinics. Furthermore, many ED patients lack access to local fall prevention clinics, and generic discharge instructions often lack the location-specific guidance that older adults need to access services close to home.

This paper describes the pre-evaluation development of an EHR-embedded intervention that automates fall risk notification and referral using a health system–integrated chatbot (ie, a virtual conversational agent). The intervention directs high-risk patients to free or low-cost, evidence-based fall prevention programs that are localized to their geographic area. Designed to scale across EDs and integrate easily into clinical workflows, this approach aims to reduce clinician burden, improve patient outcomes, and serve as a model for embedding scalable digital health interventions into routine emergency care.

## Methods

### Overview of Intervention Design

To create the intervention, we first developed a fall prevention resource library of evidence-based interventions available in the state. We then collaborated with our health system's Epic team, Livi chatbot team, and ED clinical teams to automate fall risk notification and referral to the Livi chatbot to provide location-specific fall prevention resources. Finally, we optimized the Livi chatbot intervention by incorporating feedback and experiences from end users. Each stage is described in detail in the subsequent sections.

### Ethical Considerations

The Colorado Multiple Institutional Review Board approved this study and granted exempt status (COMIRB; 24-1835). The phases described in this paper did not require patient data or consent. Community members who participated in the usability testing did not provide any identifying information.

### Fall Prevention Resources

Our team compiled a list of current fall prevention resources in the state by searching online, calling identified programs, and gathering the current resources that EDs in our system provide to patients. Resources included information about local evidence-based fall prevention classes (eg, Stepping On), and how to access help with home modifications (eg, Volunteers of America Handyperson Program)*.* We consulted the National Council on Aging's approved list of evidence-based programs for structured class and program resources. We included only free or low-cost resources to ensure broad accessibility. Our health care system's legal and compliance departments vetted these resources to ensure that there were no Stark law [10] violations. This law specifies that patient referrals must be based on patient needs rather than clinician profit. Because our health system encompasses many regions in the state, we grouped resources by location, including the Denver Metro area, Colorado's North region (eg, Fort Collins and Steamboat Springs), and the South region (eg, Colorado Springs).

### Collaboration With Key Partners

#### Industry Partner

Our health care system is in partnership with Avaamo's generative artificial intelligence (AI) conversational platform to deliver a self-service patient experience called Livi. Livi can help patients by answering clinical and logistical questions, refilling prescriptions, resetting passwords, and performing other Epic EHR-based tasks. The team responsible for deploying the Livi chatbot at our health system selected our team to conduct a pilot project that extends Livi's capabilities for ED patients.

The Avaamo platform includes advanced generative AI-based natural language understanding. This enables computers to better understand the meaning and intent of human language from patients. Within the Avaamo platform, the Livi team created intents and responses for fall prevention topics, then trained the platform by feeding it sample queries (eg, “Where can I find fall prevention resources?”) and contextual clues (eg, cities and health care specialties). In the version we developed, the Livi chatbot did not use its full large language model (LLM) capabilities. Instead, we limited it to providing rule-based answers to common fall prevention questions and specific resources that were reviewed and selected by our team. Future versions of the intervention are likely to deploy the full range of Livi's capabilities, but we opted to deploy a more limited version at this proof-of-concept stage to reduce the risk of “hallucinations” and to better control the accuracy and appropriateness of the content provided to patients.

#### Health System and Clinical Partners

We collaborated with our health system's EHR team (the ED-designated Epic team) and the Livi chatbot team to create the new notification and referral workflow. Creating an automated link to Livi in the after-visit summary (AVS) of older adult patients at high fall risk required communication and approval from the EHR and ED operations teams. Our team was the first within the health system to suggest an automated notification and referral process in the ED. This process required review and approval by the ED vice chair of clinical operations, consultation with the IT service management team, and coordination of the development, testing, and planning for the rollout of the EHR change. This coordination, from the initial planning to the launch, occurred over the course of 11 months. It involved synchronous meetings with multiple teams (ED-designated Epic team, Livi chatbot team, and ED clinical operations) that were scheduled as needed for each stage of development and occurred approximately every 3 months. Collaboration between meetings occurred approximately monthly via email with task lists and regular updates.

### User Input and Usability

We used rapid usability testing and an iterative design with end users during the development process. This user-centered design method involves gathering real-time feedback during the design process and making quick adjustments to improve functionality and experience [11,12]. Engaging end users early in the prototyping process enhances usability, ensures alignment with user needs, and increases adoption rates [13,14]. This approach aligns with implementation science frameworks that emphasize user engagement and iterative adaptation to optimize intervention effectiveness [15,16].

We engaged 93 users across 3 community events (known as research roadshows) to test usability and elicit suggestions for refinement. These sessions were held at a religious organization, an assisted living facility, and an academic medical campus in the greater Denver area. Participants included older adults, caregivers, and experts in aging and fall prevention. Using their own devices, participants scanned the QR code and navigated the Livi chatbot modules. They were encouraged to think aloud while interacting with the intervention, providing real-time feedback on its usability [17]. We also solicited specific suggestions for improvement, which guided multiple iterative updates to the Livi chatbot before implementation.

## Results

### Livi Chatbot Intervention Workflow

#### Screening for Fall Risk—Routine Care

**Figure 1:**
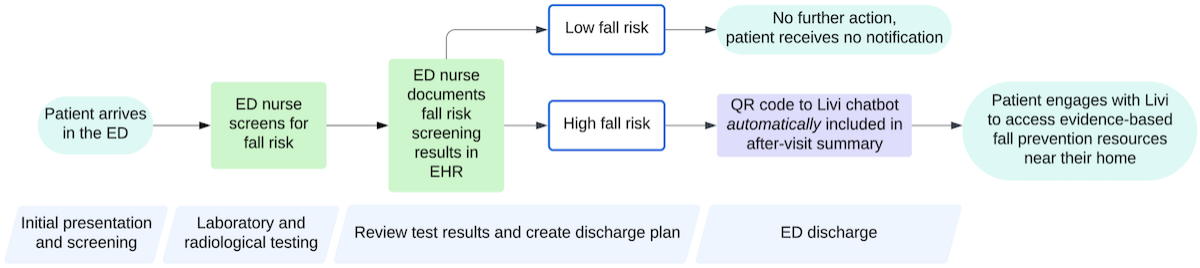
Flowchart illustrating fall risk screening and Livi chatbot resource referral within the emergency department (ED) workflow. EHR: electronic health record.

The workflow incorporating Livi is illustrated in Figure 1. In this workflow, the ED nurse first administers a fall risk screen and documents the results in the EHR nursing flow sheet as part of routine care. This fall risk screen used is the health system's Geriatric Fall Risk Score (8 questions; total possible score range, 0-24), as shown in Multimedia Appendix 1.

#### Notification of Risk and Fall Resource Referral—Livi Intervention

**Figure 2:**
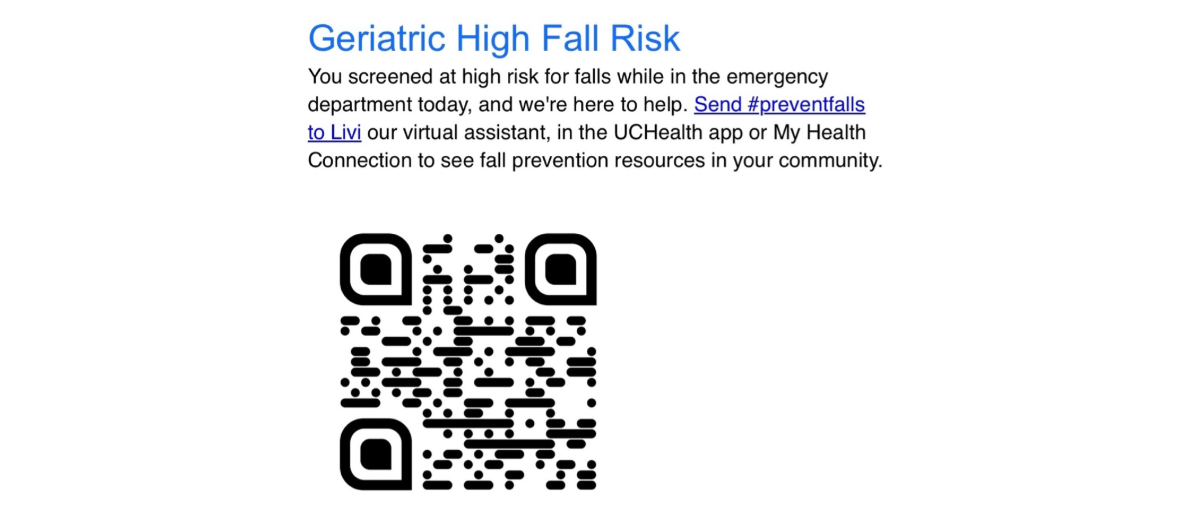
Screenshot of a message that appears in the after-visit summary for emergency department patients who screened at high fall risk. The QR code directs the patient to the Livi chatbot for fall prevention.

In the intervention, only those patients who score ≥3 (high fall risk) receive a standardized fall risk message in their AVS indicating that they screened at high fall risk and are directed to scan a QR code linking them to Livi. The AVS is printed for every patient discharged from the ED. Figure 2 illustrates the communication provided to the patient within the AVS. The AVS can be viewed by the patient in the patient portal, and the patient also receives a paper version upon ED discharge.

Patients deemed at low risk (score<3) do not receive this communication in their AVS. This process is fully automated and capitalizes on the existing ED screening results in the nursing flow sheet. The automated process reduces the reliance on clinicians to inform the patient of their fall risk and to remember to insert fall prevention referrals into the AVS.

### Livi

**Figure 3:**
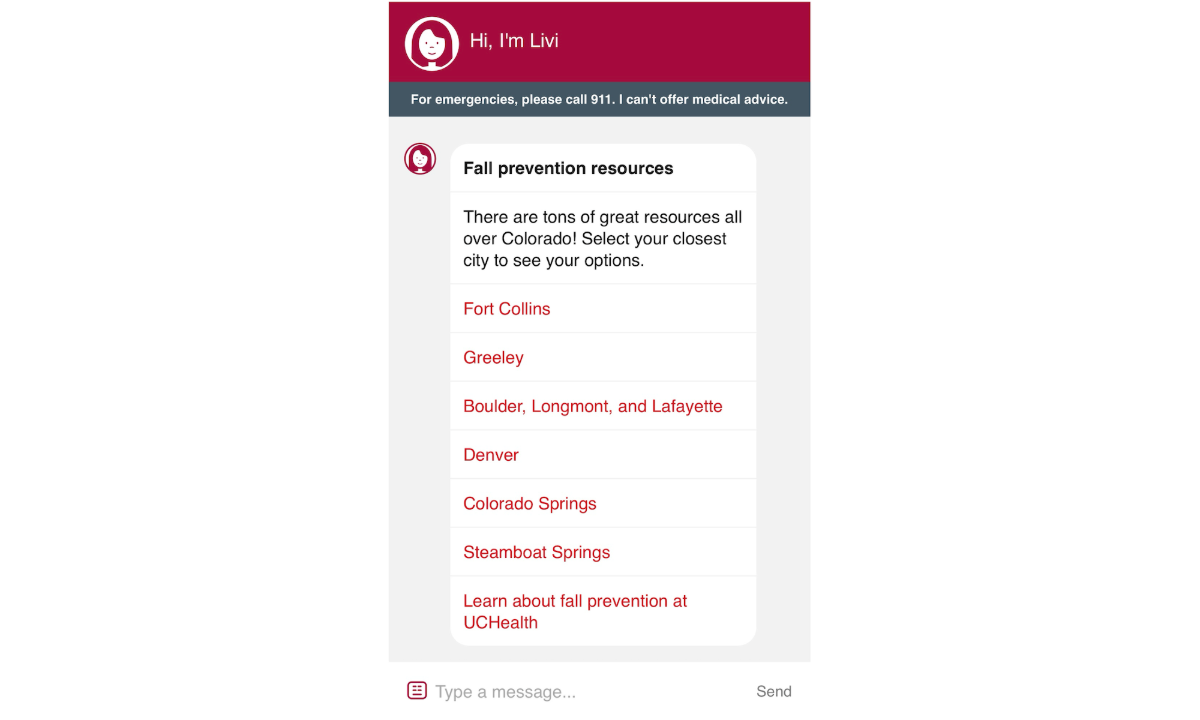
Screenshot of the Livi chatbot displaying location options. Patients select the area closest to their home, after which Livi displays fall prevention resources available in that area.

**Figure 4:**
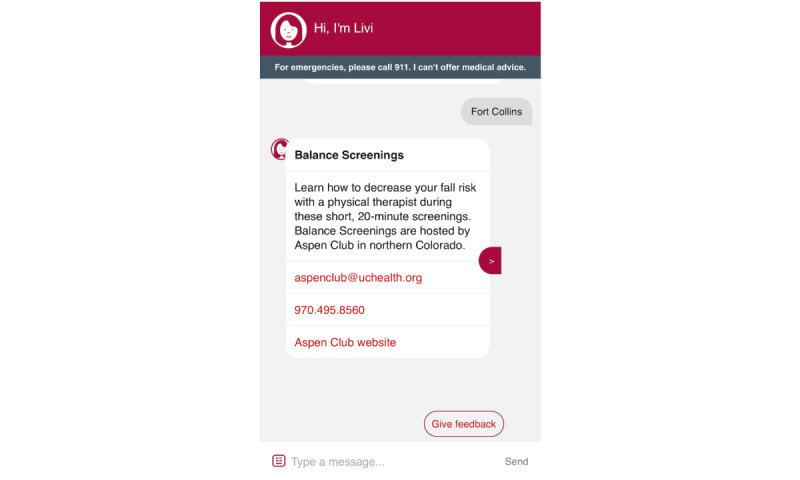
Screenshot of the Livi chatbot displaying a fall prevention resource available in the area selected by the patient (Fort Collins). The Livi chatbot describes the resource and contact information (including an email address and phone number), as well as a link to the organization’s website.

**Figure 5:**
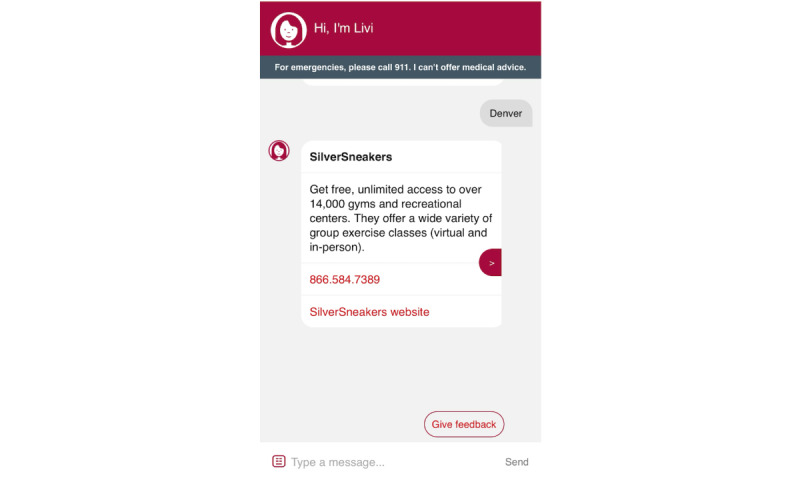
Screenshot of the Livi chatbot displaying a fall prevention resource available in the area selected by the patient (Denver). The Livi chatbot provides a description of the resource and contact information (including a phone number) and a link to the organization’s website.

**Figure 6:**
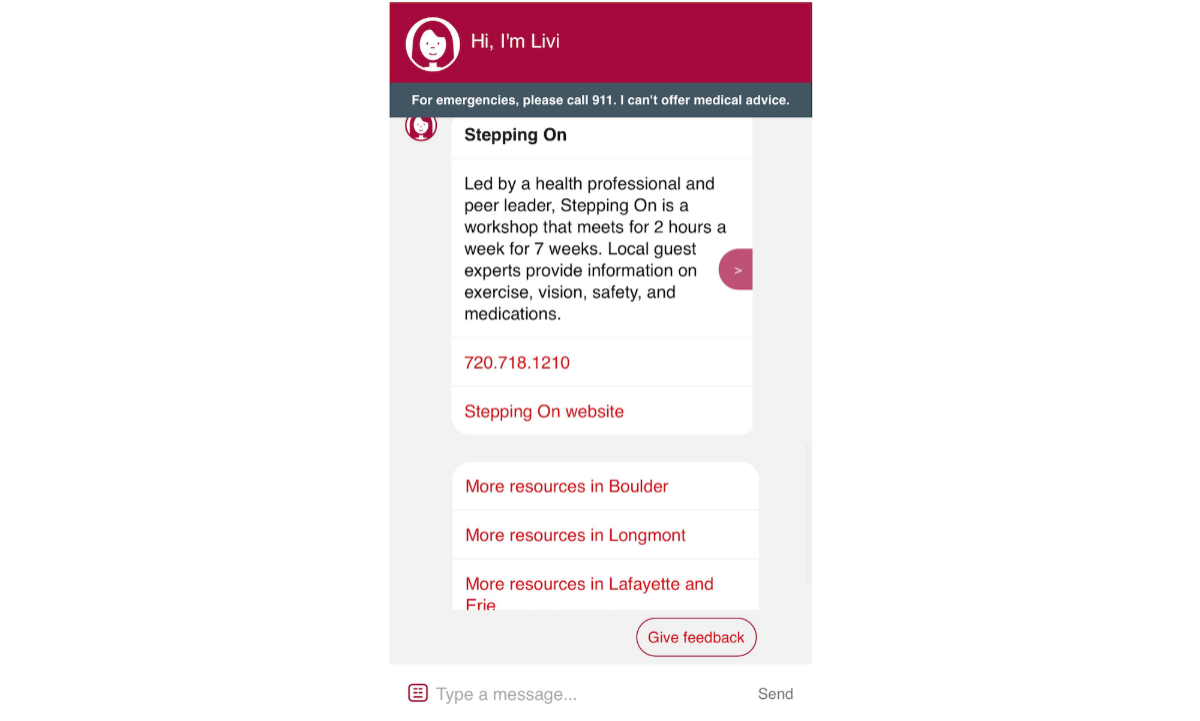
Screenshot of the Livi chatbot displaying a fall prevention resource. The Livi chatbot describes the resource and contact information (including a phone number), and a link to the organization’s website. In this example, the geographic area contains several resources (Boulder, Longmont, and Lafayette); therefore, Livi provides additional options to display resources by specific region. The option for the patient to provide feedback on Livi fall prevention is displayed in the bottom right corner.

**Figure 7:**
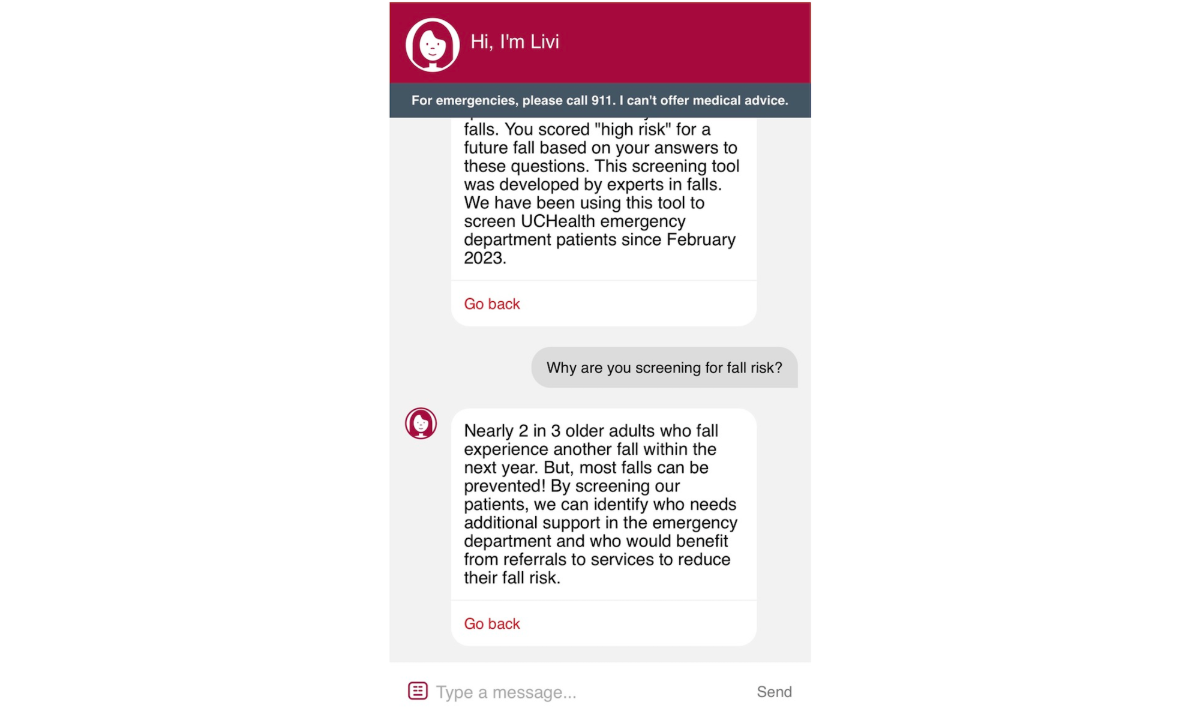
Screenshot of the Livi chatbot displaying information on the fall risk screening used in the emergency department, and additional information in response to a patient's question about fall risk screening.

Screenshots of the Livi chatbot are shown in Figures 3-7. To connect patients with appropriate resources, Livi first prompts users to select the city closest to them (Figure 3). On the basis of this selection, the chatbot provides a curated list of local fall prevention resources, each accompanied by a brief description and contact information, including an email address, phone number, and website (Figures 4-6). In addition to location-specific recommendations, Livi can answer general questions about fall risk through interactive chat responses or user-selected options to learn more (Figure 7). Patients are also given the opportunity to provide feedback on the resources offered (Figures 4-6).

The final version of Livi was refined through rapid iterative feedback, as described subsequently. For this study, we did not use Livi's natural language understanding capabilities to initiate conversations. Instead, patients accessed Livi directly through a QR code or link that launched the Livi fall prevention dialogue. From there, structured button-based navigation guided the users through tailored conversation branches based on their selections.

### Usability Testing—Iterative Design

The Livi chatbot underwent multiple iterations based on feedback from potential users (Textbox 1). Participants suggested several enhancements, including increasing font size, alternative language options, expanding geographic coverage across Colorado, and adding content on medications that increase fall risk, home modification resources, self-assessment tools, and answers to frequently asked questions about falls. Although some older adults experienced difficulty accessing Livi using the QR code, most expressed their support for the intervention.

In response, our team implemented several updates: we increased the font size, enabled language selection between English and Spanish, expanded resource coverage to include northern and southern Colorado, and incorporated the National Council on Aging 13-question fall risk self-assessment tool. Additional improvements included fall prevention tips and functionalities that allowed users to provide feedback directly within Livi.

In Table 1, we list several example questions that users could choose, along with responses from the Livi chatbot.

**Recommended modifications to Livi from usability testing with community members, and the resultant changes implemented.**

**Modifications suggested**

- Larger font
- Spanish language
- More locations
- Medication risks
- Home modification resources
- Ability to self-assess fall risk
- Definition of “high fall risk”

**Changes implemented**

- Increased font size
- Enabled a Spanish version
- Added Colorado Springs and Steamboat Springs
- Currently, we are developing a plan to incorporate information about medications and fall risk into Livi
- Added “fall prevention tips” linking to a community health pamphlet on making the home safer
- Added “check my risk for falls” linking to the National Council on Aging validated fall risk survey
- Added a statement explaining how patients were screened for fall risk during their emergency department visit (Table 1)

**Table 1:** Example questions patients may ask the Livi chatbot and corresponding responses.

Prompt	Livi chatbot’s response
What does “high fall risk” mean?	During today’s emergency department visit, nurses asked you questions to determine your risk for falls. You scored “high risk” for a future fall based on your answers to these questions. This screening tool was developed by experts in fall risk and prevention. We have been using this tool to screen UCHealth emergency department patients since February 2023.
Why are you screening for fall risk?	Nearly 2 in 3 older adults who fall experience a recurrent fall within the next year, but most falls can be prevented! By screening our patients, we can identify who needs additional support in the emergency department and who would benefit from referral to services to reduce their fall risk.
What can I do to reduce my risk?	Select the city nearest to you to see fall prevention resources in your community. These services were vetted by fall prevention experts, and we only recommend programs proven to reduce your fall risk. You may also receive brochures with specific fall prevention classes, referrals to home or outpatient physical therapy, community paramedic home-based referrals, or home health aide referrals from our staff.
What is UCHealth’s goal for fall prevention?	We hope to reduce repeat falls and injuries and empower our patients to start a dialogue about their fall risk with their primary care provider. Because we screen you for fall risk, we want to ensure you have the tools to prevent falls after you leave our emergency departments.

### Livi Go-Live

After incorporating modifications from iterative testing at community events, we launched Livi in all 17 EDs associated with our health care system (November 2024). We communicated the planned launch date, the purpose of the intervention, and shared a link to Livi with nursing staff. We also disseminated information about Livi by posting flyers in high-traffic areas within the ED. The flyer is included in Multimedia Appendix 1. We encouraged ED nurses to point out the QR code and assist patients with accessing Livi at the point of ED discharge.

### Implementation Barriers and Facilitators

We identified several informatics barriers during implementation. For example, PDF links within Livi would not open on Android devices and required reconfiguration of the fall-related resources. We also encountered challenges in integrating the QR code into the AVS, requiring collaboration with additional teams (acute and emergency business analytics) to automate Livi within the AVS of the EHR.

Facilitators of Livi implementation included the fact that all EDs within the health system use the same version of Epic, allowing the intervention to be rapidly implemented at all sites. Using a QR code that links to Livi, we can rapidly update information about the fall prevention programs when new information becomes available (eg, new contact number or location), ensuring that the information provided to patients remains timely and accurate.

## Discussion

### Principal Findings

We developed and implemented a novel digital health intervention that leverages existing fall risk screening in the ED to automate both patient notification and referral to community-based prevention resources. This approach addresses a critical gap in current care processes and exemplifies how digital tools can translate routine clinical data into automated, actionable insights. Early and ongoing engagement with end users during the design process—including older adults, caregivers, and community partners—was essential to ensure the intervention’s accessibility, usability, and relevance.

This initiative required collaboration among clinical, operational, and informatics teams. Although not all health systems have invested in chatbot infrastructure, this experience highlights a replicable model: integrating validated screeners with localized, vetted resources and embedding the pathway into standard ED workflows. By sharing our process, we aim to support other health systems in developing contextually appropriate, scalable solutions for digital referral.

Iterative usability testing demonstrated a strong community interest in the Livi chatbot and surfaced critical design improvements, such as the need for a larger font size, multilingual options, and alternate access pathways. Offering multiple modes of entry (eg, QR code in AVS, access through the electronic patient portal, or clinical staff drawing attention to the intervention) may be essential to maximizing reach and reducing disparities in access due to the digital divide, whereby older adults may not have the same digital health literacy as younger populations.

ED visits are increasingly recognized as opportunities to improve population health by screening patients for health conditions [18-21]. Automating notification to patients about their high-risk status and referring them to resources could reduce reliance on health care workers at the point of care to remember to refer eligible patients and may improve efficiency. Such digital solutions are crucial, as they may also reduce the health system’s exposure to legal, ethical, financial, and reputational risk when high-risk patients are not properly notified of their health risks and referred. Digital interventions embedded in EHR systems are recommended to expedite the integration of evidence-based interventions into practice [22].

### Comparison to Prior Work

Prior studies have provided evidence that digital interventions can be used to successfully refer patients to outpatient or specialty services [23,24], enhance patient activation [25], and help address social determinants of health [26]. Our automated screening and referral intervention shares a few commonalities with other fall risk interventions in the acute care setting. One study found that automated referral to inpatient physical therapy based on routine mobility screenings was effective in reducing 30-day readmission and death [27]. Our intervention differs in that it targets ED patients who are discharged, and it refers patients to outpatient resources using an interactive tool, Livi. Another study also used fall risk screening questions to identify patients’ fall risk and made clinicians aware of the patient’s risk status with an EHR-embedded alert. The alert included language that explained why the patient was considered at risk, and allowed the clinician to select a button to order a referral to a fall clinic within their health care system [9]. Unlike our intervention, this solution interrupts the clinician and requires them to take action to place the referral. Our intervention does not interrupt or require action from the clinician, and it refers patients to free or low-cost community resources rather than a specialty clinic. Our automated intervention that connects patients to community resources not affiliated with the hospital system may be more easily implemented and adopted in other EDs. Further study is necessary to determine whether referral to a specific fall clinic may be more effective for fall prevention than a personalized referral that allows patients to peruse and select from local resources.

Other benefits of the Livi chatbot include its automatic linkage to the AVS of high fall risk ED patients, allowing patients to return to the chatbot using their AVS printout or through their online patient portal. Settings on a patient’s phone or tablet, such as accessibility settings (eg, magnification or zoom in, bold font, screen reader capabilities, and color and contrast settings), allow the Livi information to be presented in a way that they are used to seeing and interacting with. Finally, an additional advantage of Livi is its ability to quickly update resource information, thereby ensuring that the information remains current.

### Limitations

There are limitations to this work that may impact its scalability and effectiveness. First, less technologically advanced health systems may lack the capability to operationalize the intervention. Our health system may be unique in that it has a virtual health center, a central telehealth command center, with specific resources dedicated to deploying novel EHR-embedded interventions across the health system’s Epic instance [28]. For hospitals lacking this capability, possible adaptations could include clinicians initiating the pathway to a linked website based on screening results and making fall prevention resources available in a website format rather than a chatbot. This would add steps to the clinician workflow and may be less user-friendly, but could still enable fall risk screening and prevention resources to reach high-risk patients. Other health systems with limited technological abilities may choose to develop resources that can automatically be appended to the AVS in PDF format, consistent with more typical discharge paperwork. Second, the intervention is algorithmic and requires manual programming of responses rather than taking full advantage of the potential of LLMs, which offer more natural conversation dialogue capabilities. Third, patients still need to engage with the Livi chatbot and its resources to benefit from fall prevention programs. We recognize that the adaptability and impact of the intervention are highly dependent on patients’ digital literacy and device access. Fourth, free and low-cost community-based fall prevention programs alone may not be enough to prevent falls; more personalized approaches may be needed in some cases that involve paid services, such as occupational therapy-led home safety evaluations and modifications, physical therapy services, or pharmacist-led medication reconciliation and management.

### Future Directions

We would like to expand Livi’s conversational capacity using AI while maintaining the validity of its output and full confidentiality of protected health information. The current Livi build is designed to algorithmically provide answers to common fall-related questions and vetted fall prevention resources for patients. The introduction of additional LLM features to Livi could aid in the conversational style, but these would need to be carefully balanced to prevent the incorrect output of information or “hallucinations.” Although older patients have been found to trust the health care system chatbot privacy [29], communicating patient privacy protections remains a priority, especially as expanded capabilities and chatbot access to data are being considered.

Looking ahead, we plan to expand Livi’s language options and continue enhancing its usability and feasibility for both older adults and ED staff. We are training nurses to demonstrate AVS use and plan to leverage available caregivers to help older adults use these tools. We aim to improve navigation by allowing users to browse resources by category, such as exercise programs, home modifications, and support for fear of falling, to better match individual needs. To further support continuity of care, we intend to incorporate a template that helps patients initiate a conversation with their primary care physician about fall risk. This feature could empower patients to share their screening results and more easily engage in preventive care discussions.

Finally, we will assess the effectiveness of the intervention in a future randomized controlled trial, evaluating its impact on clinical outcomes, including fall prevention behaviors, fall-related knowledge, and fall incidence. We will also collect more detailed use data and patient perspectives on the follow-up use of resources. We will gather demographic data on users to better understand who the tool is most useful for and how to further adapt it to increase scalability and inclusivity. We will measure implementation outcomes using the reach, effectiveness, adoption, implementation, and maintenance framework [30]. Our planned trial of Livi will include important clinical and usability outcomes, such as reduction in recurrent ED visits for falls, rates of participant attendance at community fall prevention programs, and likelihood of recommending the tool to others. These findings will inform future enhancements and guide broader implementation.

### Conclusions

This digital intervention offers a pragmatic, scalable solution to the longstanding gap in emergency care, ensuring that older adults identified as having a high fall risk are screened, notified, and connected to relevant, accessible fall prevention resources. By automating this process through existing clinical workflows, we aim to reduce the burden on frontline clinicians while advancing equity, efficiency, and population health. Our model is readily adaptable to other health systems seeking to transform screening into meaningful action, setting the stage for the broader implementation of digital tools that bridge acute care with community-based prevention.

## Appendix

Multimedia Appendix 1 The geriatric fall risk assessment tool and flyer used to inform emergency department staff about Livi.

## Abbreviations

AI: artificial intelligence
AVS: after-visit summary
ED: emergency department
EHR: electronic health record
LLM: large language model
