# Supplementary material for: Development and Health System Deployment of an Electronic Health Record–Integrated Chatbot Intervention for Connecting Fall Risk Screening to Community Resources After Emergency Department Visits: Implementation Study
Source: JMIR Form Res. 2025 Nov 18;9:e77237. doi: 10.2196/77237 (PMC12673308; doi:10.2196/77237)

*Supplementary File 1.* Geriatric Fall Risk Assessment Tool. Scores greater than or equal to 3 indicate high fall risk.

| *Geriatric Fall Risk Assessment Tool* |  |
| --- | --- |
| 1. Have you had 2 or more falls in the past 12 months? | Yes=3; No=0; Unable to assess=6 |
| 2. Is patient confused or disoriented? | Yes=5; No=0; Unable to assess=6 |
| 3. Is patient intoxicated or sedated? | Yes=3; No=0; Unable to assess=6 |
| 4. Do you feel unsteady when you are walking? | Yes=1; No=0; Unable to assess=6 |
| 5. Have you been told you should use a cane, walker or other assistance device to get around safely? | Yes=1; No=0; Unable to assess=6 |
| 6. Is patient experiencing altered elimination? | Yes=1; No=0; Unable to assess=6 |
| 7. Do you take more than 6 medications? | Yes=2; No=0; Unable to assess=6 |
| 8. Do you feel lightheaded or dizzy when walking? | Yes=2; No=0; Unable to assess=6 |

*Supplementary File 2.* Flyer used to inform ED staff about Livi.
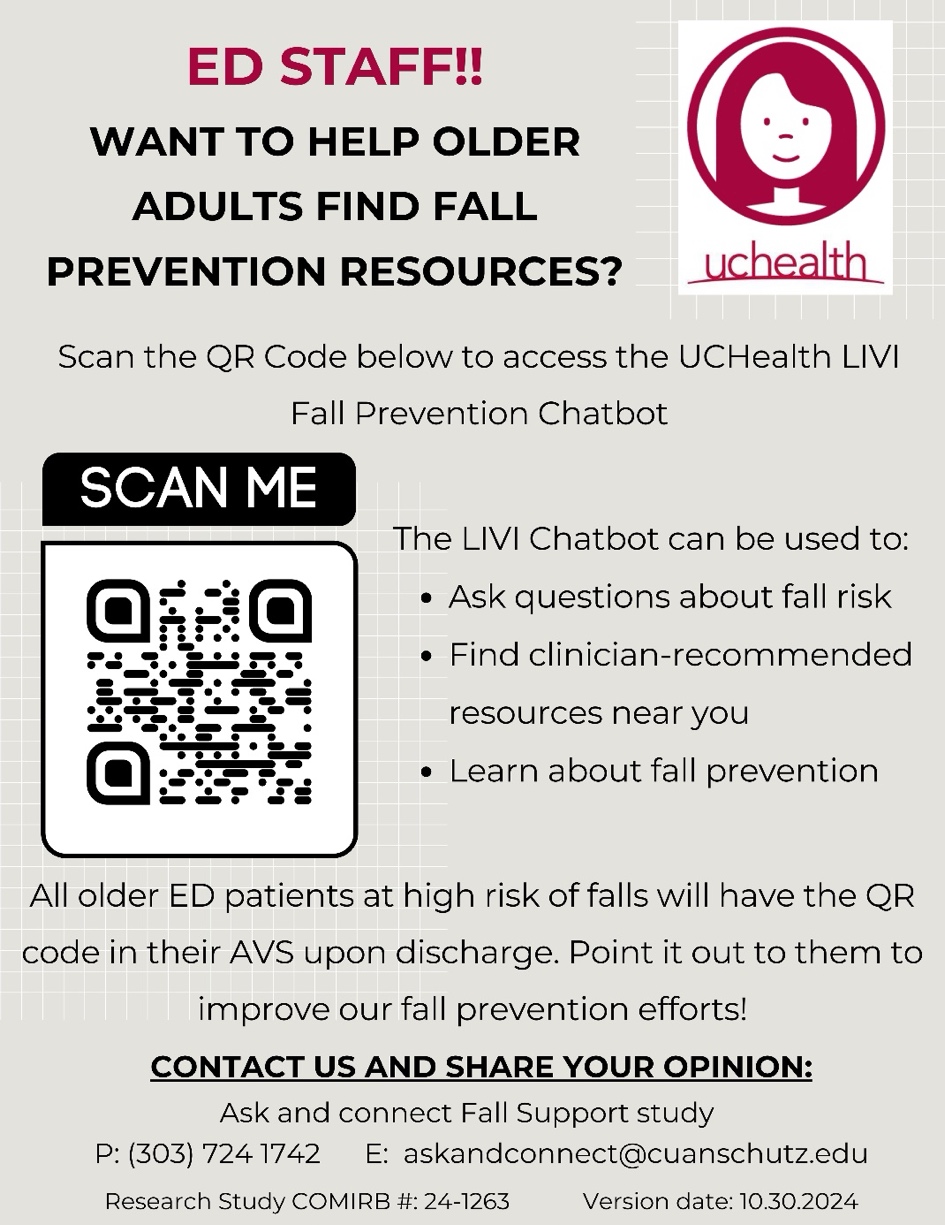

Supplement: Multimedia Appendix 1 [file formative_v9i1e77237_app1.docx]
